# Genesis Analysis of High-Gamma Ray Sandstone Reservoir and Its Log Evaluation Techniques: A Case Study from the Junggar Basin, Northwest China

**DOI:** 10.1155/2013/848201

**Published:** 2013-09-02

**Authors:** Liang Wang, Zhiqiang Mao, Zhongchun Sun, Xingping Luo, Yong Song, Zhen Liu

**Affiliations:** ^1^State Key Laboratory of Petroleum Resources and Prospecting, China University of Petroleum, Beijing 102249, China; ^2^College of Geophysics and Information Engineering, China University of Petroleum, Beijing 102249, China; ^3^Research Institute of Exploration and Development, Xinjiang Oilfield Company, PetroChina, Karamay 834000, China; ^4^No. 203 Research Institute of Nuclear Industry, Shanxi 712000, China

## Abstract

In the Junggar basin, northwest China, many high gamma-ray (GR) sandstone reservoirs are found and routinely interpreted as mudstone non-reservoirs, with negative implications for the exploration and exploitation of oil and gas. Then, the high GR sandstone reservoirs' recognition principles, genesis, and log evaluation techniques are systematically studied. Studies show that the sandstone reservoirs with apparent shale content greater than 50% and GR value higher than 110API can be regarded as high GR sandstone reservoir. The high GR sandstone reservoir is mainly and directly caused by abnormally high uranium enrichment, but not the tuff, feldspar or clay mineral. Affected by formation's high water sensitivity and poor borehole quality, the conventional logs can not recognize reservoir and evaluate the physical property of reservoirs. Then, the nuclear magnetic resonance (NMR) logs is proposed and proved to be useful in reservoir recognition and physical property evaluation.

## 1. Introduction

The exploration of complex lithological reservoir, especially the high GR reservoir, has become the frontier of oil and gas reservoir exploration [1]. Currently, high GR reservoirs are widely found in many basins all over the word, such as the western Anadarko basin [2, 3], Ordos basin northwest China [4–10], and Hailar basin northeast China [11]. Good oil and gas exploration results are obtained in these reservoirs. Therefore, high GR reservoirs have attracted geophysicists' great attention.

In 2011, a considerable number of high GR sandstone reservoirs were found in the Toutunhe Formation of Jurassic in Fudong area of Junggar basin, northwest China. Industrial oil and gas flows with high yields were obtained in these reservoirs. On the basis of GR log analysis alone, these high GR sandstone reservoirs were routinely interpreted as mudstone nonreservoirs, with negative implications for exploration and exploitation of these oil and gas reservoirs. Therefore, in order to improve the efficiency and accuracy of oil and gas exploration in these areas, it is necessary to formulate principles to distinguish high GR sandstone reservoirs from conventional sandstone reservoirs, make clear the genesis of these high GR sandstone reservoirs, and search effective log evaluation techniques for them.

## 2. Principles of Distinguishing High GR Reservoirs from Conventional Reservoirs

At present, there are no exact principles of distinguishing high GR sandstone reservoirs from conventional sandstone reservoirs. Li et al. [4] and Zhang et al. [5] take the sandstone reservoir with GR value similar to the value of mudstone as high GR sandstone reservoir, but they do not point out specific GR value criteria for high GR sandstone reservoirs. According to the GR value's differences between high GR sandstone reservoir and conventional sandstone reservoir, Zhao et al. [6] and Yu et al. [11] propose that the specific GR value criteria for high GR sandstone reservoir are 150API and 100API, respectively. They regard sandstone reservoirs with GR values exceeding these specific GR value criteria as high GR sandstone reservoirs.

It is a common law that mudstones have higher GR values and conventional sandstones have relatively lower GR values in clastic rock formations. Therefore, the GR log is widely used to calculate apparent shale content for clastic rock. Based on the principles of rock naming, sandstone and mudstone can be distinguished by the apparent shale content cut-off value of 50%. However, according to the principles of GR log, the GR value only reflects the rock's radioactive character. Thus, some sandstone reservoirs with relatively high radioactive features are often found to be symbiotic of conventional sandstone reservoirs. In order to distinguish conventional sandstone reservoir from high GR sandstone reservoir, the sandstone reservoirs with apparent shale content greater than the cut-off value of 50% are defined as high GR sandstone reservoirs. According to the GR log characters of conventional sandstone and mudstone in the study area, when the GR value is 110API, the apparent shale content is approximated to be 50%. Thus, in the study area the specific GR value criterion for high GR reservoir is 110API.

## 3. Genesis of High GR Sandstone Reservoirs

**Figure 1:**
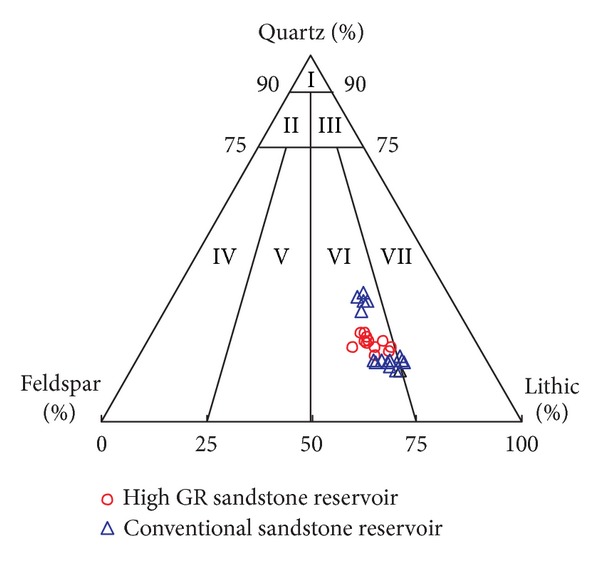
The lithology chart: I quartz sandstone, II feldspar quartz sandstone, III lithic quartz sandstone, IV feldspar sandstone, V lithic feldspar sandstone, VI feldspar lithic sandstone, and VII lithic sandstone.

**Figure 2:**
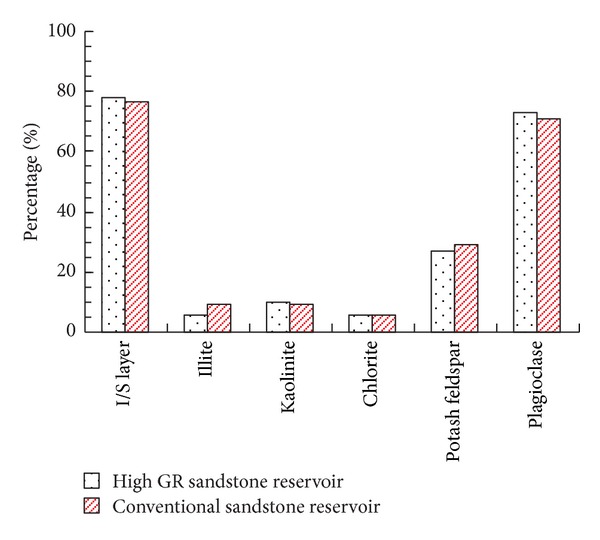
The feldspar and clay mineral types for high GR sandstone reservoir and conventional sandstone reservoir.

The feldspar, clay minerals, and tuff in sandstones usually contain or absorb radioactive elements, so the accumulation of feldspar, clay minerals, and tuff will lead to high GR character for sandstones [4–11]. In order to determine whether feldspar, clay minerals, and tuff are the direct causes for high GR sandstone reservoirs, core samples are selected from conventional sandstone reservoirs and high GR sandstone reservoirs simultaneously. Laboratory experiments, such as rock thin section analysis, X-ray diffraction analysis, and whole rock analysis, are processed on these core samples. Comparisons of experimental results between conventional sandstone reservoirs and high GR sandstone reservoirs show that these two kinds of sandstones are feldspathic lithic sandstones (Figure 1). The tuff contents (Table 1), clay minerals contents and types, and feldspar contents and types (Figure 2 and Table 1) are almost the same in high GR sandstone reservoirs and conventional sandstone reservoirs, which indicates that the tuff, feldspar, and clay minerals are not the main and direct causes for high GR sandstone reservoirs.

**Figure 3:**
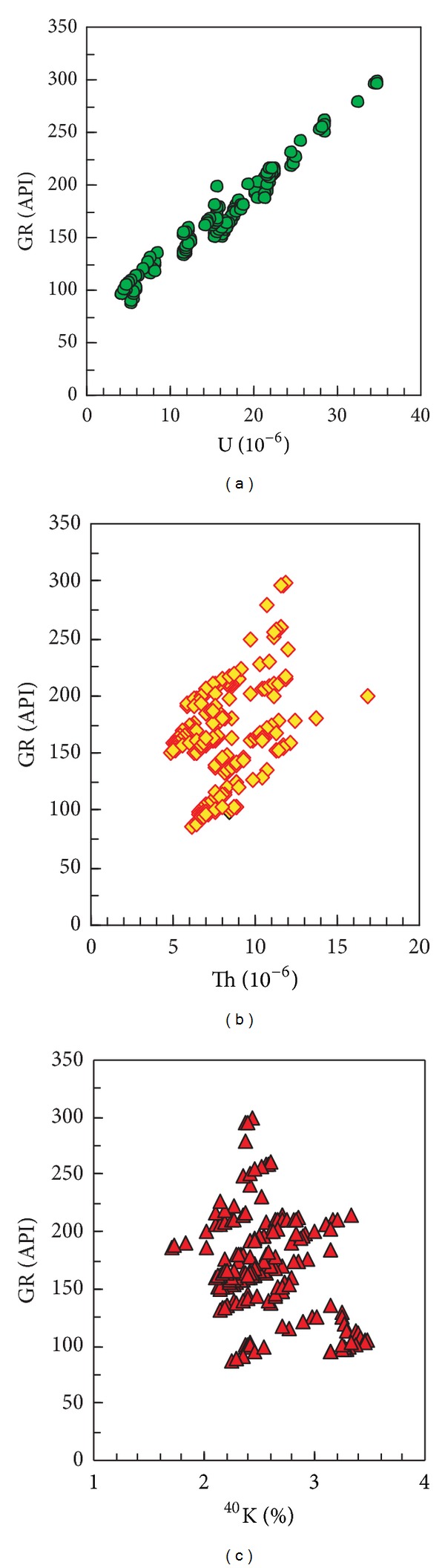
Cross plots of GR value versus (U), thorium (Th), and potassium (^40 ^K), respectively.

The GR value is the integrated response of uranium (U), thorium (Th), and potassium (^40^K) in formation [12]. The GR spectrum log reflects not only the GR value but also the uranium, thorium, and potassium content. Based on the GR spectrum log, cross plots of GR value versus uranium, thorium, and potassium are made and shown in Figure 3. Figure 3 shows that the GR value has a good positive correlation with uranium (Figure 3(a)), but poor correlations with thorium and potassium (Figures 3(b) and 3(c)). The comparisons of uranium, thorium, and potassium contents between high GR sandstone reservoirs and conventional sandstone reservoirs indicate that the uranium content in high GR sandstone reservoirs is significantly higher than that in conventional sandstone reservoirs, and the thorium and potassium contents show little difference between these two types of reservoirs (Table 1). Based on the analysis above, it can be demonstrated that the uranium enrichment is the main and direct genesis for high GR sandstone reservoir.

## 4. Genesis of Uranium Enrichment

In the period of Jurassic, a lot of tuff produced by strong volcano activities was carried by air and water to the study area and ultimately deposited with the normal sedimentary clastic particles [13]. Rock thin section analysis demonstrates that the tuff is well developed in sandstones, with an average content of 45%. The tuff with a high uranium content provides abundant uranium source to the uranium enrichment [1, 5–7, 11].

Since the beginning of the late Jurassic, the climate was arid and semiarid in the study area [14]. In arid and semi-arid climate, the soil and diving layer contain little organic content and thin humus layer, which ensures that the oxygen in formation water will not be deoxidized by the organic and humus layer in the process of formation water migration [14–20]. During oxygen bearing formation water migrating and leaching uranium bearing tuff, the U^+4^ can be oxidized to U^+6^ and uranium element transports in the form of UO_2_^+2^.

The Toutunhe Formation is composed of thick permeable sandstones interbedded with impermeable mudstone. This lithology and lithofacies combination is favorable to the uranium enrichment. The underlying Xishanyao Formation and Badaowan Formation are composed of gray mudstone and thick coal beds. The gray mudstone and thick coal beds as the main oil and gas source rock in the study area provide abundant reduced oil and gas to the reduction of UO_2_^+2^.

The 3D seismic data of this area shows that a series of small faults in the direction of east to west are found. In addition, stratigraphic unconformity is well developed between Toutunhe Formation and the underlying Xishanyao Formation. The faults combining with the stratigraphic unconformities provide channels for the upward migration of oil and gas.

Based on the analysis above, it can be concluded that the study area has abundant uranium source, favorable paleoclimate, and favorable lithology and lithofacies combination for uranium enrichment. In the process of oxygen bearing formation water migrating and leaching uranium bearing tuff, the U^+4^ can be oxidized to U^+6^ and uranium element transports in the form of UO_2_^+2^. In permeable sandstone saturated with uranium bearing formation water, the U^+6^ can be deoxidized into U^+4^ by oil and gas migrating through faults and stratigraphic unconformities and accumulates in the form of UO_2_ in sandstones.

## 5. Log Evaluation Techniques for High GR Sandstone Reservoirs

**Figure 4:**
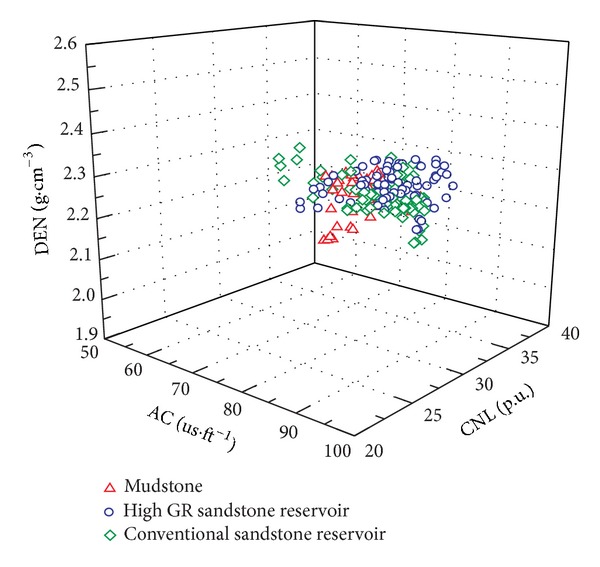
Cross plot of CNL, DEN, and AC for mudstone, conventional sandstone reservoir, and high GR sandstone reservoir.

The log evaluation for high GR sandstone reservoirs includes two aspects: reservoir recognition and physical property evaluation. In pioneering papers, the high GR sandstone reservoir can be recognized in two ways: (1) unconventional logs such as GR spectrum logs and elemental capture spectroscopy (ECS) logs can be used to recognize high GR sandstone reservoir [1, 5, 11]. But except a small amount of GR spectrum logs, there are no ECS logs in the study area. So the way of recognizing high GR reservoirs by unconventional logs is not feasible. (2) Cross plot of compensated neutron log (CNL), density log (DEN), and interval transit time log (AC) are used to identify high GR reservoir [4]. But CNL, DEN, and AC are greatly affected by formations' high water sensitivity and poor borehole quality and cannot be used to recognize high GR reservoir.  Figure 4 shows that the high GR sandstone reservoir cannot be recognized by the cross plot of CNL, DEN, and AC, because there is little difference among high GR sandstone reservoir, conventional sandstone reservoir, and mudstone in this cross plot. In this study, central type nuclear magnetic resonance (NMR) log, which is slightly or not affected by borehole quality [21], is proposed to recognize reservoirs and evaluate physical properties.

**Figure 5:**
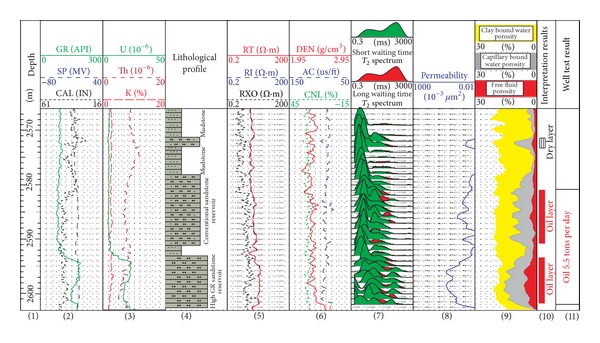
Conventional logs and NMR log responses of a well in study area.

It can be seen from Figure 5 that the high GR reservoirs' GR value exceeds 110API (track 2). The U curves in track 3 show that the uranium content of high GR sandstone reservoir is significantly higher than that of the conventional reservoir and mudstone. The track 4 and track 6 display that in characters of CNL, DEN, and AC, there is no significant difference among high GR sandstone reservoir, conventional sandstone reservoir, and mudstone. The NMR log in track 7 shows that, compared to the short transverse relaxation time (*T*_2_) of mudstone, the sandstone reservoir has a long *T*_2_. Therefore, the NMR log provides an effective way to recognize high GR reservoir.

**Figure 6:**
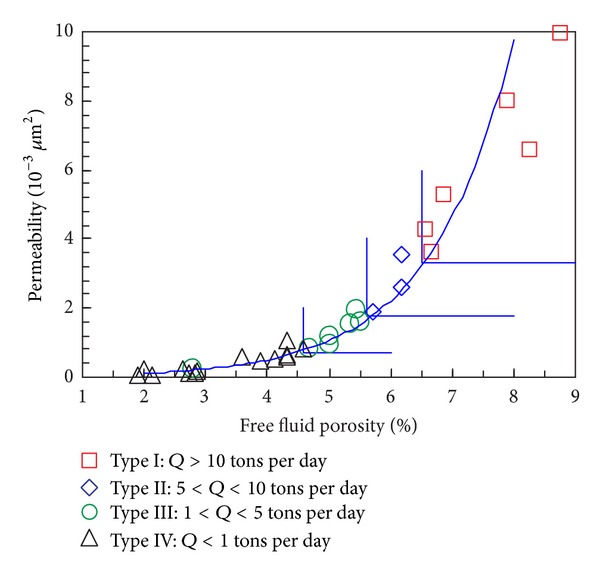
The natural fluid production capacity (*Q*) prediction chart.

The NMR log provides not only the qualitative way of identifying high GR reservoir, but also quantitative physical parameters, such as permeability, clay bound water porosity, capillary bound water porosity, and free fluid porosity (track 8 and track 9 in Figure 5). The natural fluid production capacity (*Q*) prediction chart shows that the fluid production capacity in the study area is controlled by the permeability and free fluid porosity (Figure 6). Based on the analysis above, it can be concluded that NMR log can be used to recognize high GR reservoir, evaluate reservoirs' physical properties, and even predict natural fluid production.

## 6. Conclusions

Sandstone reservoir with apparent shale content greater than 50% can be regarded as high GR sandstone reservoir. In the study area, the specific GR value criteria for high GR sandstone reservoir is 110API. 

The high GR sandstone is mainly and directly caused by abnormally high uranium enrichment, but not the tuff, feldspar, or clay mineral. The study area has abundant uranium source, favorable paleoclimate, and favorable lithology and lithofacies combination for uranium enrichment. In the oxygen bearing formation water migrating and leaching uranium bearing tuff, the U^+4^ can be oxidized to U^+6^ and uranium element transports in the form of UO_2_^+2^. In permeable sandstone saturated with uranium bearing formation water, the U^+6^ can be deoxidized into U^+4^ by oil and gas migrating through faults and stratigraphic unconformities and accumulates in the form of UO_2_ in sandstones. 

The conventional logs, which are greatly affected by formations' high water sensitivity and poor borehole quality, cannot recognize high GR sandstone reservoir and evaluate reservoirs' physical properties. NMR log proves to be useful in reservoir recognition, physical property evaluation, and even natural fluid production prediction.

## Figures and Tables

**Table 1:** The tuff content, feldspar content, clay mineral content, U, Th, and ^40^K in conventional sandstone reservoir, and high GR sandstone reservoir.

	Conventional sandstone reservoir	High GR sandstone reservoir
Tuff content (%)	45.35	44.93
Feldspar content (%)	22.58	24.71
Clay mineral content (%)	3.75	3.05
Th content (10^−6^)	7.19	8.23
U content (10^−6^)	4.47	16.08
^ 40^K content (%)	2.58	2.56
